# Tissue Microarrays to Visualize Influenza D Attachment to Host Receptors in the Respiratory Tract of Farm Animals

**DOI:** 10.3390/v13040586

**Published:** 2021-03-31

**Authors:** Nikoloz Nemanichvili, Alinda J. Berends, Richard W. Wubbolts, Andrea Gröne, Jolianne M. Rijks, Robert P. de Vries, Monique H. Verheije

**Affiliations:** 1 Department of Biomolecular Health Sciences, Division of Pathology, Faculty of Veterinary Medicine, Utrecht University, 3584 CL Utrecht, The Netherlands; n.nemanichvili@uu.nl (N.N.); A.J.Berends@uu.nl (A.J.B.); A.Grone@uu.nl (A.G.); M.H.Verheije@uu.nl (M.H.V.); 2 Department of Biomolecular Health Sciences, Division of Cell Biology, Metabolism & Cancer, Faculty of Veterinary Medicine, Utrecht University, 3584 CL Utrecht, The Netherlands; r.wubbolts@uu.nl; 3 Dutch Wildlife Health Centre, Department of Biomolecular Health Sciences, Faculty of Veterinary Medicine, Utrecht University, 3584 CL Utrecht, The Netherlands; J.M.Rijks@uu.nl; 4 Department of Chemical Biology and Drug Discovery, Utrecht Institute for Pharmaceutical Sciences, Utrecht University, 3584 CG Utrecht, The Netherlands

**Keywords:** influenza D, tissue microarray, host tropism, farm animals, 9-O-acetylated sialic acid

## Abstract

The trimeric hemagglutinin-esterase fusion protein (HEF) of influenza D virus (IDV) binds 9-O-acetylated sialic acid receptors, which are expressed in various host species. While cattle are the main reservoir for IDV, the viral genome has also been detected in domestic pigs. In addition, antibodies against IDV have been detected in other farm animals such as sheep, goats, and horses, and even in farmers working with IDV positive animals. Viruses belonging to various IDV clades circulate, but little is known about their differences in host and tissue tropism. Here we used recombinantly produced HEF proteins (HEF S57A) from the major clades D/Oklahoma (D/OK) and D/Oklahoma/660 (D/660) to study their host and tissue tropism and receptor interactions. To this end, we developed tissue microarrays (TMA) composed of respiratory tissues from various farm animals including cattle, domestic pigs, sheep, goats, and horses. Protein histochemical staining of farm animal respiratory tissue-microarrays with HEF proteins showed that cattle have receptors present over the entire respiratory tract while receptors are only present in the nasal and pharyngeal epithelium of pigs, sheep, goats, and horses. No differences in tropism for tissues and animals were observed between clades, while hemagglutination assays showed that D/OK has a 2-fold higher binding affinity than D/660 for receptors on red blood cells. The removal of O-acetylation from receptors via saponification treatment confirmed that receptor-binding of both clades was dependent on O-acetylated sialic acids.

## 1. Introduction

Influenza D (IDV) virus was first identified in 2011 and belongs to the *Orthomyxoviridae* family [1]. The virus is a single-strand, negative-sense RNA virus consisting of 7 genomic segments. Initially classified as an influenza C (ICV) strain (C/swine/Oklahoma/1334/2011), further characterization studies of the pathogen revealed it to be a distinct new virus and it was thus reclassified as influenza D (D/swine/Oklahoma/1334/2011) [2]. Serological analyses have found that IDV has a broader host tropism than ICV, as IDV circulates in farm animals such as cattle (*Bos taurus*), domestic pigs (*Sus scrofa domesticus*), sheep (*Ovis aries*), goats (*Capra aegagrus domesticus*), and horses (*Equus ferus caballus*). Humans working with IDV positive cattle and pigs have been found positive for anti-IDV antibodies [3,4,5,6,7,8]. Moreover, infection studies have confirmed that cattle, pigs, and ferrets (*Mustela putorius furo*) are susceptible to IDV infection, with cattle as the main reservoir for IDV [2,9,10,11]. No experiments have been conducted thus far to confirm the susceptibility of sheep, goats, and horses.

IDV strains can be categorized into two major clades, D/Oklahoma (D/OK) and D/660/Oklahoma (D/660), and two minor clades as D/China and D/Japan [12,13,14]. The original D/swine/Oklahoma/1334/2011 strain belongs to the D/Oklahoma clade and is the most widespread, being found in North America, Europe, Asia, Africa, and South America [3,12,15,16,17,18]. The D/660 clade was at first only isolated in North America, however, D/660 strains were recently also found in cattle herds in Ireland and Italy [3,6,19,20]. All IDV clades target the respiratory tract of their host, with rather mild clinical signs in cattle such as coughing, runny nose, and elevated temperature; no clinical signs have been observed in infected domestic pigs [13]. Differences in clinical signs between clades have thus far not been reported.

Like ICV, IDV uses 9-O-Acetylated sialic acids (9-O-Ac Sia) receptors to bind to host cells by its hemagglutinin-esterase fusion (HEF) protein [21,22]. The hemagglutinin domain attaches to host receptors while the esterase domain has a receptor-destroying function to release new viral particles, similar to the HE proteins found in the bovine coronavirus [23]. It has been hypothesized that the more open receptor-binding cavity of the IDV HEF protein accommodates a more extended range of 9-O-Ac structures compared to the receptor-binding of ICV HEF, which might explain influenza D’s broader host tropism [21,24,25,26]. The crystal structure of the binding pocket of D/660 has not yet been elucidated, so it remains unclear whether differences in receptor binding avidity exist between the two clades. While the D/OK clade attaches to both 9-O-Ac Neu5Ac and Neu5Gc sialic acids, the receptor specificity for the D/660 clade of IDV has not been revealed [3,19,27]. Besides, it is unknown whether both clades have the same tissue and host tropism.

In this study, we aimed to explore the tissue tropism of IDV D/OK and D/660 in the respiratory tract of farm animals including cattle, domestic pigs, sheep, goats, and horses. To visualize binding interactions between HEF and host cells we used recombinant IDV HEF proteins, generated using sequences from a D/OK and a D/660 strain, in tissue staining studies and hemagglutination assays. We demonstrate that using in-house developed tissue microarrays, both D/OK and D/660 HEF proteins bind to receptors present on respiratory tract tissues of a wide variety of farm animals. In cattle tissues, receptor binding was seen across the entire respiratory system for both clades, while in pigs, sheep, goats, and horses, receptor binding was only observed in tissues from the upper respiratory tract. Observed differences in binding for tissues and red blood cells between the two strains, did not result in differences in host or tissue tropism.

## 2. Materials and Methods

### 2.1. Genes, Site-Directed Mutagenesis, and Expression Vectors

Human codon-optimized HEF sequences from D/bovine/France/5920/2014 (D/OK clade, GenBank: MG720235) and D/bovine/Nebraska/9-5/2012 (D/660 clade, GenBank: KM392471) were obtained from GenScript and cloned into the pCD5 vector as previously described [28]. The resulting pCD5-HEF-GCN4-sfGFP-Strep encodes for the hemagglutinin and esterase domains of the HEF protein in frame with a trimerization domain, sfGFP and Strep-tag located C-terminally [28,29] HEF esterase knockout mutants were generated by site-directed mutagenesis (Q5, New England Biolabs, Ipswich, MA, USA) using primers introducing a single point mutation in the DNA codon changing serine at position 57 into alanine (S57A). The HA proteins of A/Vietnam/1203/04/H5N1 (GenBank: EF541403) and A/Indonesia/05/05/H5N1 (Genbank: EF541394) were used as control proteins. Both HA protein expression vectors were created as previously described [30].

### 2.2. Protein Expression and Purification

Proteins were produced using a mammalian cell culture system previously described [28]. Briefly, pCD5-HEF expression vectors were transfected into HEK293T cells (ATCC^®^ CRL-3216™) by incubation with polyethyleneimine (PEI) in a 1:10 DNA/PEI ratio for 16 h at 37 °C. The transfection reagent was then removed and replaced with medium [293 SFM II suspension medium supplemented with 2.0 g/L primatone, 3.6 g/L sodium bicarbonate, 3.0 g/L primatone (Kerry, Naas, Kildare, Ireland), 1% GlutaMax (Gibco, Waltham, MA, USA), 1.5% DMSO, and 2 mM valproic acid] with a further incubation of 5 days at 37 °C after which the supernatant was harvested. The presence of HEF protein in the supernatant was analyzed by Western blot using α-strep-tag mouse antibodies (IBA Life Sciences, Göttingen, Lower Saxony, Germany) at a 1:2000 dilution. Finally, the HEF proteins were purified using sepharose strep-tactin beads (IBA Life Sciences, Göttingen, Lower Saxony, Germany) as previously described [29]. The two control proteins A/H5 Indo and A/H5 VN Y161A were created and produced as previously described [30].

### 2.3. Tissue Collection and Tissue Microarrays Creation Using 3D Printed Array Plates

Tissues originating from the respiratory tract (nasal epithelium, pharyngeal epithelium, upper trachea, lower trachea, and lung) were collected from cattle, domestic pigs, sheep, goats, and horses. These tissues were obtained from deceased animals sent for diagnostic and educational purposes to the Veterinary Pathologic Diagnostic Center, Faculty of Veterinary Medicine, Utrecht University. Tissues were fixed in buffered formaldehyde 4% m/v for 24–48 h at room temperature after which they were transferred to a 70% ethanol solution for storage. Tissues were processed according to standard paraffin embedding procedures. Microscopic evaluation of hematoxylin and eosin-stained slides was performed to determine tissue quality before selection and punching of tissue cores for incorporation into microarrays using a 2 mm biopsy punch pen (Miltex, Plainsboro Township, NJ, USA). These tissue cores were then incorporated in an empty 3D printed array plate.

The 3D printed array plates were designed using 123D Design (Autodesk, Mill Valley, CA, USA) and printed with a Sigma Dual Extruder R17 (BCN3D Technologies, Barcelona, Barcelona, Spain) 3D printer using 2.85mm diameter PLA filament (MarkerPoint PLA, Dordrecht, South Holland, Netherlands) using BCN3D Cura (1.02b) with a 0.6 mm nozzle (205 °C, 80 % infill, wall 0.5 mm) that was z calibrated before printing. Best glass plate attachment with a skirt adhesion setting was obtained by applying 3D LAC spray (3D LAC) onto the ethanol cleaned glass plate. Samples were printed on the heated bed (65 °C) with a z resolution of 0.1 mm at lower speeds (50 mm/s) to increase accuracy. These array plates have a 6 × 6 layout to accommodate up to 36 tissue cores on a single array plate. Once the plate was filled with the required amount of tissue cores it was embedded into paraffin and left to cool. These tissue microarray blocks were then cut into thin 0.3 um slices onto microscopy slides for protein histochemical staining.

### 2.4. Hemagglutination Assay

HEF proteins were precomplexed with antibodies as described in the previous paragraph. Both RBC suspensions were washed with calcium- and magnesium-free PBS and diluted to 0.5% before incubation with 2-fold serial dilutions of the precomplexed HEF-antibody mix in 96-well V-bottom plates. Chemical inhibition of esterase activity was achieved by adding Diisopropylfluorophosphate (DFP) to the precomplexed HEF-antibody mixture to a final dilution of 2% *v*/*v* during precomplexing.

### 2.5. Protein Histochemical Staining

Tissue slides were deparaffinized and rehydrated before antigen retrieval with citric acid buffer pH 6. Nonspecific binding was blocked using 3% bovine serum albumin in a humidity chamber overnight at 4 °C. For tissue staining, the HEF protein was first precomplexed with α-strep-tag mouse HRP antibody and goat-α-mouse-HRP at a 4:2:1 ratio in PBS for 20 min on ice. This precomplexed mixture was then applied onto the tissue and incubated for 90 min at room temperature. Subsequently, the slides were washed 3 times in PBS and HEF protein binding was visualized by incubating AEC substrate for 15 min. Hematoxylin and eosin stains were performed to visualize tissue structure and morphology before covering the stained slides with coverslips using AquaTex (Merck, Kenilworth, NJ, USA).

With the fluorescent protein histochemical staining, a similar incubation procedure with precomplexed HEF protein was performed, followed by adding 50 mM DAPI for 5 min to visualize cell nuclei. Afterward, slides were washed 3 times in PBS to remove excess DAPI, and slides were covered with coverslips using FluorSave (Merck, Kenilworth, NJ, USA).

For saponification of the tissue, slides were incubated for 5 min at room temperature with 2 M NaOH pH 10 before the addition of the precomplexed HEF-antibody mix. The slides were then washed 3 times for 5 min with PBS, after which the protein histochemical staining was continued as described.

## 3. Results

### 3.1. Expression and Characterization of the Recombinant Influenza D HEF Proteins

**Figure 1:**
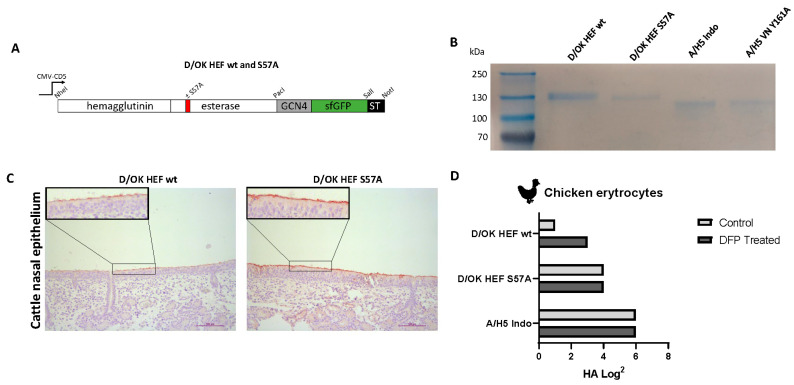
(**A**) Schematic overview of the expression plasmid used for the production of the HEF protein in a mammalian cell culture system. The ORF is controlled by a cytomegalovirus (CMV) promoter with a CD5 signal peptide for excretion of the HEF protein from the cell into the supernatant. To create D/OK HEF S57A, a single point mutation at amino acid 57 was switched from serine to alanine to knock out esterase activity. (**B**) Coomassie staining of the purified HEF proteins D/OK HEF wt and D/OK HEF S57A, and HA control proteins A/H5 Indo and A/H5 VN Y161A. (**C**) Protein histochemical staining of cattle nasal epithelium to compare protein binding between wildtype D/OK HEF wt and esterase knockout mutant D/OK HEF S57A. Scale bars are 100 µm (**D**) Hemagglutination assay for comparing protein binding avidity using chicken erythrocytes between wildtype D/OK HEF wt and esterase knockout D/OK HEF S57A. The chemical inhibitor diisopropyl fluorophosphate (DFP) was used to directly inhibit esterase activity. The HA protein A/H5 Indo was used as a negative control for its lack of esterase activity.

To study tissue tropism and receptor binding properties of influenza D viruses, we recombinantly produced two HEF proteins from D/bovine/France/5920/2014 (D/OK clade): a wildtype (D/OK HEF wildtype), and an S57A mutant in which esterase activity was eliminated (D/OK HEF S57A) (Figure 1A) [21]. Expression of both proteins in mammalian cells was confirmed by visualization of green fluorescence as previously described [28]. The correct molecular weight of both proteins was confirmed by SDS-PAGE, in which both monomers had an electrophoretic mobility corresponding to 130 kDa (Figure 1B). The biological activity of the proteins was tested by performing protein histochemical staining on cattle nasal epithelium (Figure 1C). Both proteins bind to the epithelial layer of the cattle nasal tissue, however, the HEF S57A exhibits more intense and even distribution of label, which might be due to the mutation in the esterase-active site. Next, a hemagglutination assay was performed in the presence of diisopropylfluorophosphate (DFP) which inhibits the esterase activity [21]. In the absence of DFP, the D/OK HEF wildtype protein only hemagglutinated when undiluted, while the addition of the DFP inhibitor increases HA activity 4-fold. On the other hand, the hemagglutination ability of the D/OK HEF S57A, and that of influenza A virus-derived trimeric HA proteins (A/H5 Indo), were as expected not affected by DFP. In subsequent experiments, the esterase knockout mutant S57A of this strain, and that of the D/bovine/Nebraska/9-5/2012 (D/660 clade) strain were used.

### 3.2. D/OK and D/660 Clade HEF Proteins Have Comparable Receptor Binding Avidity to Erythrocytes of Different Species

**Figure 2:**
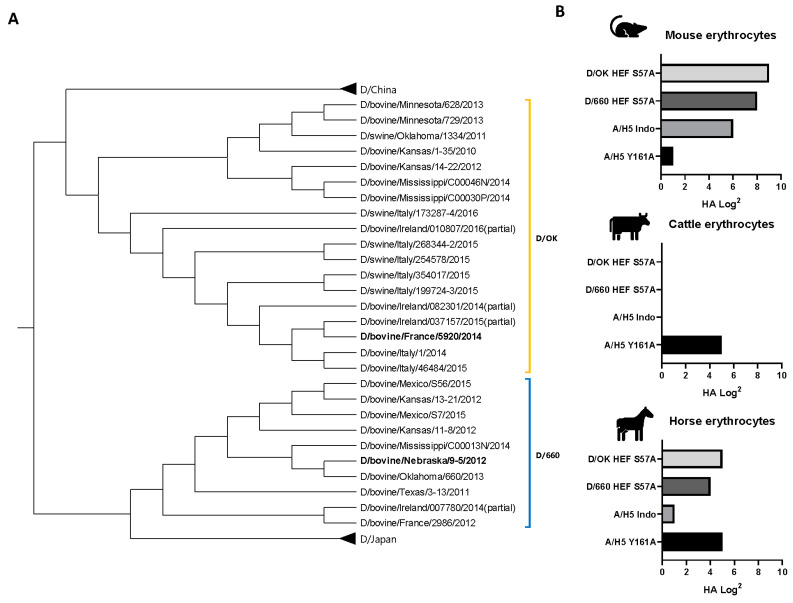
(**A**) Phylogenetic tree of influenza D HEF protein gene. Sequences are listed by country, species isolated, location, and year of isolation. The genes are clustered into D/OK or D/660 clades. The two strains used in this research have been highlighted in bold. (**B**) Hemagglutination assays comparing the protein binding avidity of both D/OK and D/660 HEF S57A proteins in mouse, cattle, and horse erythrocytes along with two H5 HA proteins A/H5 Indo and A/H5 VN Y161A as controls.

We set out to determine whether there are differences in binding avidity between the HEFs of the two clades since on a phylogenetic comparison both clades (Figure 2A) can have substantial differences in their HEF protein sequence which could affect binding avidity. The first step was to compare this in a hemagglutination assay. Using mouse erythrocytes, known to have a high percentage of 9-O-Ac Neu5Ac Sia on the surface [31,32], we demonstrate that while both HEF proteins of D/OK and D/660 have strong binding avidity, D/OK had a 2-fold higher binding avidity than D/660 (Figure 2B). Hemagglutination assays using erythrocytes from cattle, as the reservoir species for IDV but known to lack 9-O-Ac [32], reveal that neither of the proteins can bind to cattle erythrocytes. Lastly, horse erythrocytes were tested to see if both clades are capable of binding to 9-O-Ac Sia’s with an N-glycolyl (Neu5Gc) linkage [21]. Both clades displayed binding avidity on equine erythrocytes with again a 2-fold difference in favor of D/OK. The overall results are in line with the previous observation that HEF proteins are dependent on the acetylation of sialic acid receptors, with D/OK having a 2-fold higher binding avidity than D/660. To determine if this leads to a difference in host tropism, we next performed protein histochemical staining on farm animal tissues.

### 3.3. HEF Proteins Bind to Cattle Respiratory Tissues But Are Restricted towards Domestic Pig Upper Respiratory Tract Tissues

**Figure 3:**
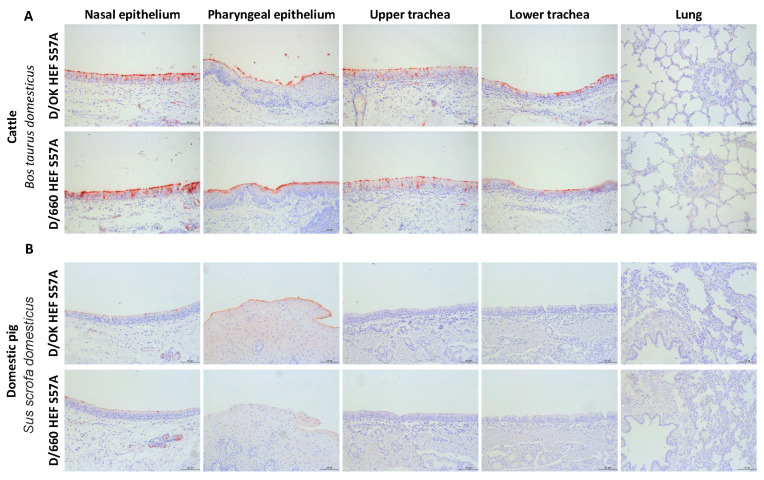
(**A**) Protein histochemical staining of tissue micro-array containing cattle (5 on array) respiratory tissues with D/OK and D/660 HEF S57A proteins at 50 µg/mL and (**B**) staining of tissue micro-array containing domestic pig (6 on array) respiratory tissues with D/OK and D/660 HEF S57A proteins at 50 µg/mL. Scale bars are 100 µm. Technical repeats were performed 3 times on both microarrays. See Supplementary Tables S1 and S2 for the content and arrangement of each microarray.

As cattle are the main reservoir for influenza D viruses, we first compared the binding properties of the two clades to respiratory tissues of this species. To this end, we developed an in-house tissue microarray of respiratory tract tissues of cattle, consisting of nasal epithelium, pharyngeal epithelium, upper trachea, lower trachea, and lung. protein histochemistry staining showed that both D/OK and D/660 HEF bound to all cattle tissues from the respiratory tract except for the lungs (Figure 3A). To test the binding and tissue tropism of both clades for domestic pig tissues a similar approach was taken. Both clades bound with a comparable tissue tropism in the upper respiratory tract nasal and pharyngeal epithelium of domestic pigs (Figure 3B), but not to the epithelial lining of any of the other tissues. Stronger binding was seen for D/OK than for D/660 on the nasal epithelium, while on the pharyngeal epithelium comparable binding between the two clades was observed. In domestic pigs, strong binding could also be seen in the submucosal glands of the upper respiratory tract tissues, a location that is known to produce mucus rich in acetylated decoy structures [33]. Overall, the results show that both clades can bind to receptors present over the entire respiratory tract of cattle while in domestic pigs binding is limited to the upper part of the respiratory tract.

### 3.4. D/660 HEF Has Higher Binding Avidity But Similar Tissue Tropism Compared to D/OK HEF

**Figure 4:**
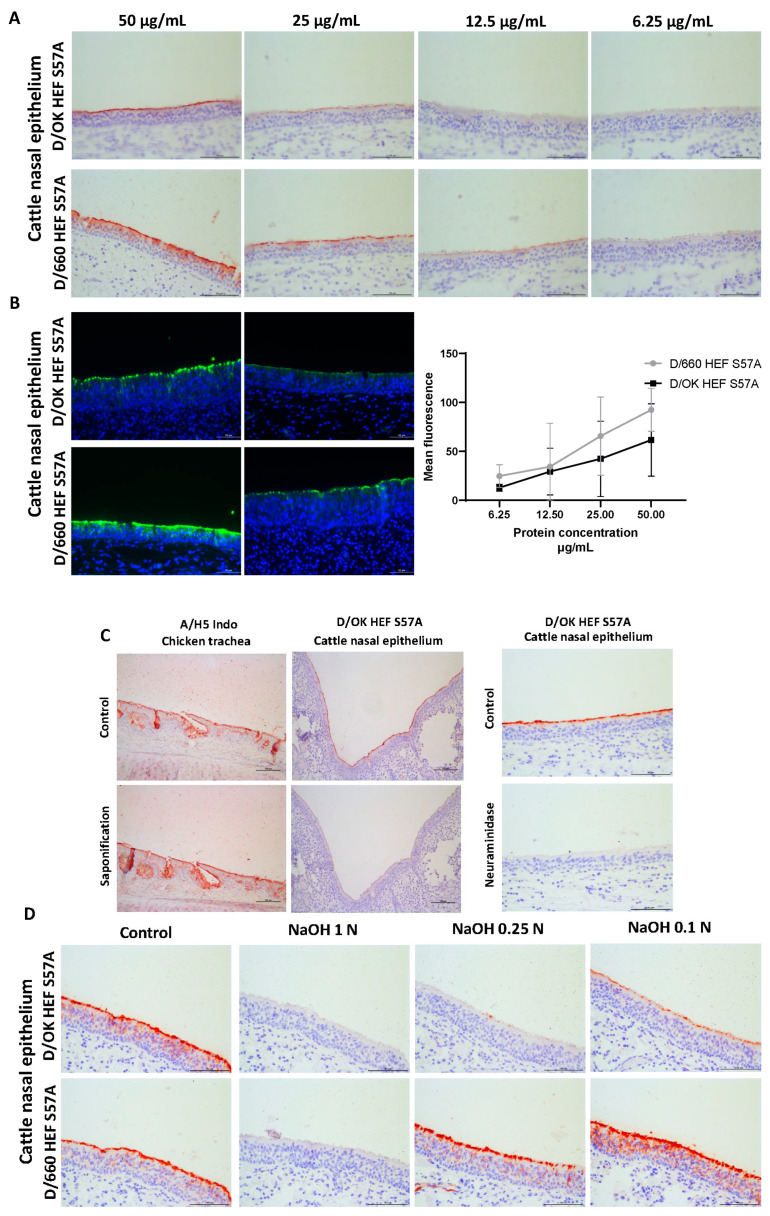
(**A**) Protein histochemical staining of cattle nasal epithelium with a concentration-dependent serial dilution of D/OK and D/660 HEF S57A protein. Technical repeats were performed 3 times. Scale bars are 100 µm (**B**) A repeat of the same serial dilution for protein fluorescent imaging for quantification of the fluorescent signal as a value for binding avidity of the HEF protein. Scale bars are 50 µm. Error bars indicate standard deviation. Technical repeats were performed 3 times. (**C**) Saponification treatment of chicken trachea and cattle nasal epithelium with NaOH to remove acetylation from sialic acid receptors and a neuraminidase treatment of cattle nasal epithelium to remove sialic acids. The A/H5 Indo HA protein was used as a negative control for acetylated sialic acid dependency while D/OK HEF S57A was used to show both acetylated sialic acid dependency in saponification and sialic acid dependency on neuraminidase treated tissue. Scale bars are 100 µm. Technical repeats were performed 2 times. (**D**) Serial dilution of NaOH normality for saponification treatment of cattle nasal epithelium tissues. Higher normality treated tissues will contain less acetylated sialic acids with the dilution series indicating the turning point for when D/OK HEF S57A and D/660 HEF S57A regain binding avidity. Scale bars are 100 µm. Technical repeats were performed 2 times.

Next, to allow a more quantitative comparison of the differences in binding avidity between the two clades, we performed a protein concentration-dependent dilution on cattle nasal epithelium. In parallel, Protein histochemical staining for visual analyses (Figure 4A) and fluorescent staining for quantification (Figure 4B) were performed. With the Protein histochemical staining (Figure 4A) it was observed that the D/OK HEF protein could be diluted until 25 µg/mL while D/660 HEF retains binding until a concentration of 12.5 µg/mL. Quantification of fluorescence (Figure 4B) showed that across all concentrations D/660 maintains a stronger fluorescent signal output than D/OK protein, indicating a stronger binding avidity than D/OK. As there are currently no methods to specifically detect 9-O-acetylated sialic acids via histochemistry, we needed another approach to confirm that the difference in binding avidity between the clades was indeed caused by binding to acetylated sialic acid receptors. To do this, we performed an additional experiment where via saponification, acetylation was removed from sialic acid present on tissues (Figure 4C) [34,35]. Saponification-treated tissues could no longer be bound by HEF.

Proteins (Figure 4D) while the A/H5 Indo HA protein, which is not dependent on acetylation for binding sialic acid remained unaffected by the treatment. A neuraminidase treatment further confirmed sialic acid dependency (Figure 4D). Finally, we performed a serial dilution of the saponification treatment by decreasing the normality (N) of the NaOH base in the buffer. Similar to in the protein dilution experiment the D/660 HEF protein regained binding avidity first at 0.25 N while the D/OK HEF protein only binds when saponification was performed in the presence of 0.1 N NaOH or lower. These results confirm that the D/660 HEF protein has a slightly higher binding avidity than D/OK, albeit with no difference in tissue tropism between the two clades.

### 3.5. D/OK and D/660 HEF Proteins Show Similar Binding and Tissue Tropism on Sheep, Goats, and Horses

**Figure 5:**
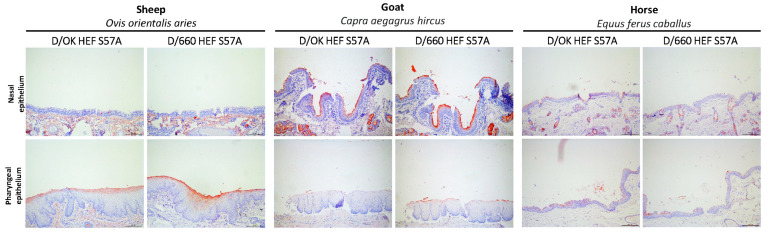
Protein histochemical staining of tissue micro-arrays containing sheep (2 on array), goat (4 on array), and horse (5 on array) respiratory tissues with D/OK and D/660 HEF S57A proteins at 50 µg/mL, only the nasal and pharyngeal epithelium is shown, see the Supplementary Figure S1 for the protein histochemical staining of the respiratory tract of sheep goat and horse. Scale bars are 20 µm. Technical repeats were performed 3 times on each microarray. See Supplementary Tables S3–S5 for content and arrangement of each microarray.

Finally, we selected tissues of farm animals that have been reported positive in serological surveys in literature. Upper respiratory tract tissues from sheep, goats, and horses were all bound by both HEF proteins (Figure 5), confirming that these species contain D/OK and D/660 HEF receptors in the upper respiratory tract. The tissue tropism for D/OK and D/660 is the same in all three species. Sheep, goat, and horses’ tissues demonstrate that HEF protein binding is restricted to the upper respiratory tract, which similar to pigs. Saponification and neuraminidase treatments on these tissues also confirmed that binding of the HEF proteins to the receptors of these host species was dependent on acetylation and that all receptors were sialic acids. Overall, the results show that like in domestic pigs both IDV clades are limited to binding receptors in the upper respiratory tract of sheep, goats, and horses.

## 4. Discussion

In this study, we generated HEF proteins of the D/OK and D/660 clades to explore the tissue and host tropism of two major IDV clades. Both esterase-inactivated HEF proteins were able to undergo receptor binding in respiratory tissues of all of the tested farm animals. In hemagglutination assays, D/OK has a slightly higher binding avidity than D/660, while on tissues, either when applying a dilution series of HEF or after saponification, D/660 rather bound with slightly higher avidity. We speculate that D/660 HEF either has stronger binding to the acetylated sialic receptors or it recognizes a wider selection of acetylated sialic acid receptors on tissues.

HEFs of both IDV clades recognize and interact with receptors expressed on cells of the cattle’s upper and lower respiratory tract, except for the lung. This was expected since the species is the main reservoir for IDV [1,4,14]. Although the hemagglutination assay results showed a difference in binding avidity between the two clades, no differences in tissue specificity were observed. In domestic pigs, we only observed receptor binding in the upper respiratory tract, namely nasal and pharyngeal epithelium. Staining was also observed in the submucosal glands, which could indicate the presence of receptors in mucus [25,36]. Pathogenesis in pigs is significantly lower compared to that in cattle, which could be due to infection being restricted to the upper respiratory tract of domestic pigs [5,13]. Another reason why IDV is much more prevalent in cattle could be because IDV has been suggested to contribute to, or even benefits from, bovine respiratory disease (BRD), as it is among the three most commonly found viral agents in dairy calves affected by BRD [4,37,38]. It might be that limitation of the infection to the upper parts of the respiratory tracts reduces complications or that BRD enhances cattle susceptibility to IDV over the entire respiratory tract, though thus far no experimental evidence for this has been provided but would also be a potential explanation as to why no binding can be seen in the lungs of cattle in this study even though multiple studies have reported finding IDV replication in lungs of infected cattle [4,39,40,41]. A controlled experimental study in cattle has confirmed that IDV can replicate in the lower respiratory tract however the clinical impact and pathogenicity were moderate compared to infections in the upper respiratory tract [42]. The lack of sufficient receptors in the lower respiratory tract might explain why IDV infection is more severe in the upper respiratory tract when compared to other respiratory viruses.

A comparison of the two clades in terms of binding avidity shows that D/660 has a higher avidity than D/OK in the hemagglutination assay, concentration-dependent serial dilution, and saponification experiments. However, the higher binding avidity of D/660 does not seem to give the clade an advantage over D/OK when it comes to host or tissue tropism as seen in the results of the protein histochemical staining on the farm animals. So far, it is thus unclear what the cause of this higher avidity for one or more particular receptors or a wider selection of acetylated sialic acid receptors is and if it is significant enough for D/660 to gain an edge over D/OK. However, the overwhelming majority of IDV reported strains are still of the D/OK clade [3,12,15,16,17,18]. Further research into this matter can be done by using artificial glycan arrays to determine 9-O-Ac receptor recognition ranges differences between the two clades and/or comparative infection studies using varying amounts of viral loads, a process which is underway as printing of 9-O-acetylated glycans is being refined [43].

The results of the protein histochemical staining on sheep, goats, and horses show that both clades can also bind to receptors in the upper respiratory tract of other farm animals. Previous research has shown that serological responses to IDV were present in these species and thus that they might be susceptible to the virus [4,27]. The evidence for the presence of receptors is provided by this study and confirms that the first requirement for a potential IDV infection exists [3]. The comparable host and tissue tropism indicate that D/660 has the same potential for binding in these species as D/OK. While no infection studies or detection of viral genomes of these species have been reported in the literature so far, more reports are coming in of IDV antibody detection in European sheep and D/660 qPCR detected in European domestic pigs [3,19,44]. A previous study successfully showed by using explants from sheep, that D/OK clade IDV replicates in the respiratory tract of sheep [6]. These results complement our findings for the D/OK clade as this study found that sheep contain receptors to which D/OK clade IDV can attach and thus has the first step set for viral infection. However, the explant study did not include a D/660 clade virus and it would be interesting to see if a D/660 IDV would match the replication results that were found for the D/OK clade strain. In particular, since the findings in this study show that D/660 clade receptor attachment is near identical in host and tissue tropism compared to D/OK clade, showing that there is a need to perform extensive replication studies in sheep, goats, and horses for both D/OK and D/660 clade IDV. In summary, this study demonstrates that the first requirement for infection, that is, the binding of the viral attachment proteins to host cells, in several farm animal species is met.

## 5. Conclusions

In conclusion, we demonstrate that all five tested farm animal species express host surface receptors for both the D/OK and the D/660 clade of IDV. While cattle tissues expressed receptors in the entire respiratory tract except for the lungs, HEF binding was restricted to the nasal and pharyngeal epithelium in domestic pigs, sheep, goats, and horses.

## Data Availability

All relevant data are within the paper and its supplementary data.

## Supplementary Materials

The following are available online at https://www.mdpi.com/article/10.3390/v13040586/s1, Figure S1: Protein histochemical staining of tissue micro-arrays containing sheep (2 on array), goat (4 on array), and horse (5 on array) respiratory tissues with D/OK and D/660 HEF S57A proteins at 50 µg/mL on the upper and lower respiratory tract, Table S1: Composition and arrangement of the cattle (Bos taurus domesticus) tissue microarray, Table S2: Composition and arrangement of the domestic pig (Sus scrofa domesticus) tissue microarray, Table S3: Composition and arrangement of the sheep (Ovis orientalis aries) tissue microarray, Table S4: Composition and arrangement of the goat (Caora aegagrus hircus) tissue microarray, Table S5: Composition and arrangement of the horse (Equus ferus caballus) tissue microarray.

## References

[1] Hause B.M., Ducatez M., Collin E.A., Ran Z., Liu R., Sheng Z., Armien A., Kaplan B., Chakravarty S., Hoppe A.D. (2013). Isolation of a novel swine influenza virus from Oklahoma in 2011 which is distantly related to human influenza C viruses. PLoS Pathog..

[2] Hause B.M., Collin E.A., Liu R., Huang B., Sheng Z., Lu W., Wang D., Nelson E.A., Li F. (2014). Characterization of a novel influenza virus in cattle and Swine: Proposal for a new genus in the Orthomyxoviridae family. mBio.

[3] O’Donovan T., Donohoe L., Ducatez M.F., Meyer G., Ryan E. (2019). Seroprevalence of influenza D virus in selected sample groups of Irish cattle, sheep and pigs. Ir. Vet. J..

[4] Ferguson L., Olivier A.K., Genova S., Epperson W.B., Smith D.R., Schneider L., Barton K., McCuan K., Webby R.J., Wan X.F. (2016). Pathogenesis of Influenza D Virus in Cattle. J. Virol..

[5] Ferguson L., Luo K., Olivier A.K., Cunningham F.L., Blackmon S., Hanson-Dorr K., Sun H., Baroch J., Lutman M.W., Quade B. (2018). Influenza D Virus Infection in Feral Swine Populations, United States. Emerg. Infect. Dis..

[6] Mazzetto E., Bortolami A., Fusaro A., Mazzacan E., Maniero S., Vascellari M., Beato M.S., Schiavon E., Chiapponi C., Terregino C. (2020). Replication of Influenza D Viruses of Bovine and Swine Origin in Ovine Respiratory Explants and Their Attachment to the Respiratory Tract of Bovine, Sheep, Goat, Horse, and Swine. Front Microbiol.

[7] White S.K., Ma W., McDaniel C.J., Gray G.C., Lednicky J.A. (2016). Serologic evidence of exposure to influenza D virus among persons with occupational contact with cattle. J. Clin. Virol..

[8] Nedland H., Wollman J., Sreenivasan C., Quast M., Singrey A., Fawcett L., Christopher-Hennings J., Nelson E., Kaushik R.S., Wang D. (2018). Serological evidence for the co-circulation of two lineages of influenza D viruses in equine populations of the Midwest United States. Zoonoses Public Health.

[9] Sreenivasan C., Thomas M., Sheng Z., Hause B.M., Collin E.A., Knudsen D.E., Pillatzki A., Nelson E., Wang D., Kaushik R.S. (2015). Replication and Transmission of the Novel Bovine Influenza D Virus in a Guinea Pig Model. J. Virol..

[10] Holwerda M., Kelly J., Laloli L., Stürmer I., Portmann J., Stalder H., Dijkman R. (2019). Determining the Replication Kinetics and Cellular Tropism of Influenza D Virus on Primary Well-Differentiated Human Airway Epithelial Cells. Viruses.

[11] Liu R., Sreenivasan C., Yu H., Sheng Z., Newkirk S.J., An W., Smith D.F., Chen X., Wang D., Li F. (2020). Influenza D virus diverges from its related influenza C virus in the recognition of 9-O-acetylated N-acetyl- or N-glycolyl-neuraminic acid-containing glycan receptors. Virology.

[12] Luo J., Ferguson L., Smith D.R., Woolums A.R., Epperson W.B., Wan X.F. (2017). Serological evidence for high prevalence of Influenza D Viruses in Cattle, Nebraska, United States, 2003–2004. Virology.

[13] Yu J., Li F., Wang D. (2020). The first decade of research advances in influenza D virus. J. Gen. Virol.

[14] Collin E.A., Sheng Z., Lang Y., Ma W., Hause B.M., Li F. (2015). Cocirculation of two distinct genetic and antigenic lineages of proposed influenza D virus in cattle. J. Virol..

[15] Salem E., Cook E.A.J., Lbacha H.A., Oliva J., Awoume F., Aplogan G.L., Hymann E.C., Muloi D., Deem S.L., Alali S. (2017). Serologic Evidence for Influenza C and D Virus among Ruminants and Camelids, Africa, 1991–2015. Emerg. Infect. Dis..

[16] Zhai S.L., Zhang H., Chen S.N., Zhou X., Lin T., Liu R., Lv D.H., Wen X.H., Wei W.K., Wang D. (2017). Influenza D Virus in Animal Species in Guangdong Province, Southern China. Emerg. Infect. Dis..

[17] Horimoto T., Hiono T., Mekata H., Odagiri T., Lei Z., Kobayashi T., Norimine J., Inoshima Y., Hikono H., Murakami K. (2016). Nationwide Distribution of Bovine Influenza D Virus Infection in Japan. PLoS ONE.

[18] Ducatez M.F., Pelletier C., Meyer G. (2015). Influenza D virus in cattle, France, 2011–2014. Emerg. Infect. Dis..

[19] Orla F., Clare G., Jean M., Claire I., Mariette D., Ben H., Guy M., Eoin R. (2018). Influenza D Virus in Cattle, Ireland. Emerg. Infect. Dis. J..

[20] Ferguson L., Eckard L., Epperson W.B., Long L.P., Smith D., Huston C., Genova S., Webby R., Wan X.F. (2015). Influenza D virus infection in Mississippi beef cattle. Virology.

[21] Song H., Qi J., Khedri Z., Diaz S., Yu H., Chen X., Varki A., Shi Y., Gao G.F. (2016). An Open Receptor-Binding Cavity of Hemagglutinin-Esterase-Fusion Glycoprotein from Newly-Identified Influenza D Virus: Basis for Its Broad Cell Tropism. PLoS Pathog..

[22] Pleschka S., Klenk H.-D., Herrler G. (1995). The catalytic triad of the influenza C virus glycoprotein HEF esterase: Characterization by site-directed mutagenesis and functional analysis. J. Gen. Virol..

[23] Mayr J., Haselhorst T., Langereis M.A., Dyason J.C., Huber W., Frey B., Vlasak R., de Groot R.J., von Itzstein M. (2008). Influenza C virus and bovine coronavirus esterase reveal a similar catalytic mechanism: New insights for drug discovery. Glycoconj J..

[24] Wang M., Veit M. (2016). Hemagglutinin-esterase-fusion (HEF) protein of influenza C virus. Protein Cell.

[25] Wasik B.R., Barnard K.N., Ossiboff R.J., Khedri Z., Feng K.H., Yu H., Chen X., Perez D.R., Varki A., Parrish C.R. (2017). Distribution of O-Acetylated Sialic Acids among Target Host Tissues for Influenza Virus. mSphere.

[26] Harms G., Reuter G., Corfield A.P., Schauer R. (1996). Binding specificity of influenza C-virus to variablyO-acetylated glycoconjugates and its use for histochemical detection ofN-acetyl-9-O-acetylneuraminic acid in mammalian tissues. Glycoconj. J..

[27] Quast M., Sreenivasan C., Sexton G., Nedland H., Singrey A., Fawcett L., Miller G., Lauer D., Voss S., Pollock S. (2015). Serological evidence for the presence of influenza D virus in small ruminants. Vet Microbiol.

[28] Nemanichvili N., Tomris I., Turner H.L., McBride R., Grant O.C., van der Woude R., Aldosari M.H., Pieters R.J., Woods R.J., Paulson J.C. (2018). Fluorescent Trimeric Hemagglutinins Reveal Multivalent Receptor Binding Properties. J. Mol. Biol..

[29] de Vries R.P., de Vries E., Bosch B.J., de Groot R.J., Rottier P.J.M., de Haan C.A.M. (2010). The influenza A virus hemagglutinin glycosylation state affects receptor-binding specificity. Virology.

[30] Broszeit F., Tzarum N., Zhu X., Nemanichvili N., Eggink D., Leenders T., Li Z., Liu L., Wolfert M.A., Papanikolaou A. (2019). N-Glycolylneuraminic Acid as a Receptor for Influenza A Viruses. Cell Rep..

[31] Rogers G.N., Herrler G., Paulson J.C., Klenk H.D. (1986). Influenza C virus uses 9-O-acetyl-N-acetylneuraminic acid as a high affinity receptor determinant for attachment to cells. J. Biol. Chem..

[32] Barnard K.N., Alford-Lawrence B.K., Buchholz D.W., Wasik B.R., LaClair J.R., Yu H., Honce R., Ruhl S., Pajic P., Daugherity E.K. (2020). Modified Sialic Acids on Mucus and Erythrocytes Inhibit Influenza A Virus Hemagglutinin and Neuraminidase Functions. J. Virol..

[33] Reuter G., Pfeil R., Stoll S., Schauer R., Kamerling J.P., Versluis C., Vliegenthart J.F.G. (1983). Identification of new Sialic Acids Derived from Glycoprotein of Bovine Submandibular Gland. Eur. J. Biochem..

[34] Volz D., Reid P.E., Park C.M., Owen D.A., Dunn W.L. (1987). Histochemical procedures for the simultaneous visualization of neutral sugars and either sialic acid and its side chain O-acyl variants or O-sulphate ester. I. Methods based upon the selective periodate oxidation of sialic acids. Histochem. J..

[35] Phillips A.D. (2004). Acetylated sialic acid residues and blood group antigens localise within the epithelium in microvillous atrophy indicating internal accumulation of the glycocalyx. Gut.

[36] Barnard K.N., Wasik B.R., LaClair J.R., Buchholz D.W., Weichert W.S., Alford-Lawrence B.K., Aguilar H.C., Parrish C.R. (2019). Expression of 9-O- and 7,9-O-Acetyl Modified Sialic Acid in Cells and Their Effects on Influenza Viruses. mBio.

[37] Ng T.F., Kondov N.O., Deng X., Van Eenennaam A., Neibergs H.L., Delwart E. (2015). A metagenomics and case-control study to identify viruses associated with bovine respiratory disease. J. Virol..

[38] Dane H., Duffy C., Guelbenzu M., Hause B., Fee S., Forster F., McMenamy M.J., Lemon K. (2019). Detection of influenza D virus in bovine respiratory disease samples, UK. Transbound Emerg. Dis..

[39] Mosier D. (2014). Review of BRD pathogenesis: The old and the new. Anim. Health Res. Rev..

[40] Zhang X., Outlaw C., Olivier A.K., Woolums A., Epperson W., Wan X.F. (2019). Pathogenesis of co-infections of influenza D virus and Mannheimia haemolytica in cattle. Vet. Microbiol.

[41] Lee J., Wang L., Palinski R., Walsh T., He D., Li Y., Wu R., Lang Y., Sunwoo S.-Y., Richt J.A. (2019). Comparison of Pathogenicity and Transmissibility of Influenza B and D Viruses in Pigs. Viruses.

[42] Salem E., Hagglund S., Cassard H., Corre T., Naslund K., Foret C., Gauthier D., Pinard A., Delverdier M., Zohari S. (2019). Pathogenesis, Host Innate Immune Response, and Aerosol Transmission of Influenza D Virus in Cattle. J. Virol..

[43] Khedri Z., Xiao A., Yu H., Landig C.S., Li W., Diaz S., Wasik B.R., Parrish C.R., Wang L.P., Varki A. (2017). A Chemical Biology Solution to Problems with Studying Biologically Important but UnsTable 9-O-Acetyl Sialic Acids. ACS Chem. Biol..

[44] Chiapponi C., Faccini S., Fusaro A., Moreno A., Prosperi A., Merenda M. (2019). Detection of a New Genetic Cluster of Influenza D Virus in Italian Cattle. Viruses.

