# Supplementary material for: Tissue Microarrays to Visualize Influenza D Attachment to Host Receptors in the Respiratory Tract of Farm Animals
Source: Viruses. 2021 Mar 31;13(4):586. doi: 10.3390/v13040586 (PMC8067312; doi:10.3390/v13040586)
Supplement: Supplementary file 1 [file viruses-13-00586-s001.zip › Supplementary Figure S1.pdf]

Supplementary Figure S1

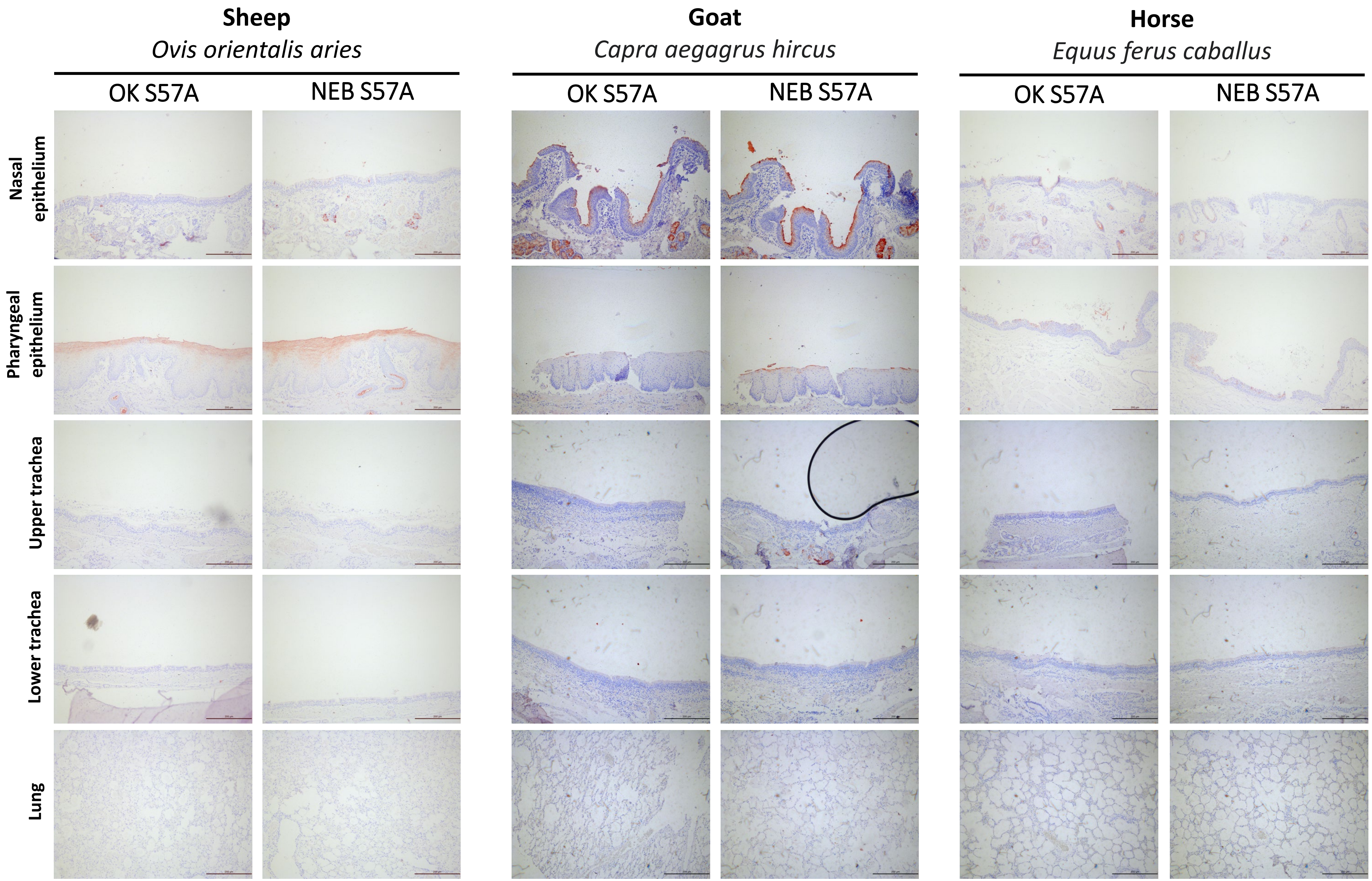

Figure S1. Protein histochemical staining of tissue micro-arrays containing sheep (2 on array), goat (4 on array), and horse (5 on array) respiratory tissues with D/OK and D/660 HEF S57A proteins at 50 µg/ml on the upper and lower respiratory tract.
