# Supplementary material for: Tissue Microarrays to Visualize Influenza D Attachment to Host Receptors in the Respiratory Tract of Farm Animals
Source: Viruses. 2021 Mar 31;13(4):586. doi: 10.3390/v13040586 (PMC8067312; doi:10.3390/v13040586)
Supplement: Supplementary file 1 [file viruses-13-00586-s001.zip › Supplementary Table S1, S2, S3, S4 & S5.pdf]

Table S1: Composition and arrangement of the cattle (*Bos taurus domesticus*) tissue microarray.

	**A – Cattle 1** 3190705039	**B – Cattle 2** 3190124023	**C – Cattle 3** 3190508048	**D – Cattle 4** 3190703001	**E – Cattle 5** 3190703030	**F**
**1**	Nasal epithelium	Nasal epithelium	Nasal epithelium	Nasal epithelium	Nasal epithelium	x
**2**	Pharynx	x	Pharynx	Pharynx	Pharynx	x
**3**	Upper trachea	Upper trachea	Upper trachea	Upper trachea	Upper trachea	x
**4**	Lower trachea	Lower trachea	Lower trachea	Lower trachea	Lower trachea	x
**5**	Primary Bronchus	x	Primary Bronchus	Primary Bronchus	Lung	x
**6**	Lung	Lung	Lung	Lung	Primary Bronchus	x

Table S2: Composition and arrangement of the domestic pig (*Sus scrofa domesticus*) tissue microarray.

	**A – Dom. Pig 1** 3190502038	**B – Dom. Pig 2** 3190509021	**C – Dom. Pig 3** 3191010028	**D – Dom. Pig 4** 3191010025	**E – Dom. Pig 5** 3191010026	**F – Dom. Pig 5** 3191010027
**1**	Nasal epithelium	Nasal epithelium	Nasal epithelium	Nasal epithelium	Nasal epithelium	Nasal epithelium
**2**	Pharynx	Pharynx	Pharynx	x	x	Pharynx
**3**	Upper trachea	Upper trachea	Upper trachea	Upper trachea	Upper trachea	Upper trachea
**4**	Lower trachea	Lower trachea	Lower trachea	Lower trachea	Lower trachea	Lower trachea
**5**	Primary Bronchus	Primary Bronchus	Primary Bronchus	Primary Bronchus	Primary Bronchus	Lung
**6**	Lung	Lung	Lung	Lung	x	x

Table S3: Composition and arrangement of the sheep (*Ovis orientalis aries*) tissue microarray.

	**A**	**B – Sheep 1** 3190503010	**C**	**D – Sheep 2** 3190612024	**E**	**F**
**1**	x	Nasal epithelium	x	Nasal epithelium	x	x
**2**	x	Pharynx	x	Pharynx	x	x
**3**	x	Upper trachea	x	Upper trachea	x	x
**4**	x	Lower trachea	x	Lower trachea	x	x
**5**	x	Prim. Bronchus	x	Prim. Bronchus	x	x
**6**	x	Lung	x	Lung	x	x

Table S4: Composition and arrangement of the goat (*Caora aegagrus hircus*) tissue microarray.

	**A**	**B – Goat 1** 3181003029	**C – Goat 2** 3191024026	**D – Goat 3** 60899/2018	**E – Goat 4** 1104026	**F**
**1**	x	Nasal epithelium	Nasal epithelium	Nasal epithelium	Nasal epithelium	x
**2**	x	Pharynx	Pharynx	Pharynx	Pharynx	x
**3**	x	Upper trachea	Upper trachea	Upper trachea	Upper trachea	x
**4**	x	Lower trachea	Lower trachea	Lower trachea	Lower trachea	x
**5**	x	Prim. Bronchus	Prim. Bronchus	Prim. Bronchus	Lung	x
**6**	x	Lung	Lung	Lung	Prim. Bronchus	x

Table S5: Composition and arrangement of the horse (*Equus ferus caballus*) tissue microarray.

	**A – Horse 1** 3191126005	**B – Horse 2** 3190128026	**C – Horse 3** 320010029	**D – Horse 4** 3200113001	**E – Horse 5** 3160226033	**F**
**1**	Nasal epithelium	Nasal epithelium	Nasal epithelium	Nasal epithelium	Nasal epithelium	x
**2**	Pharynx	Pharynx	x	Pharynx	Pharynx	x
**3**	Upper trachea	Upper trachea	Upper trachea	Upper trachea	Upper trachea	x
**4**	Lower trachea	Lower trachea	Lower trachea	x	Upper trachea	x
**5**	Primary Bronchus	Primary Bronchus	Primary Bronchus	x	Prim. Bronchus	x
**6**	Lung	Lung	Lung	Lung	x	x

The number under each animal indicates the general laboratory information management system (GLIMS) number under which the animal is registered at Faculty of Veterinary Medicine, Utrecht University. X's mark locations where no tissue is located.

All arrays were checked using plant lectins MAL and SNA for presence of sialic acid receptors as quality control. D/OK HEF S57A and D/660 HEF S57A binding controls to acetylated-sialic acids were confirmed via neuraminidase treatment to confirm sialic acid dependence and via saponification for acetylated sialic acid dependence. All controls and technical repeats were performed 3 times per tissue microarray. No anomalies or variances were detected in any of the controls or repeats.
